# DIRECT trial. Diverticulitis recurrences or continuing symptoms: Operative versus conservative Treatment. *A MULTICENTER RANDOMISED CLINICAL TRIAL*

**DOI:** 10.1186/1471-2482-10-25

**Published:** 2010-08-06

**Authors:** Bryan JM van de Wall, Werner A Draaisma, Esther CJ Consten, Yolanda van der Graaf, Marten H Otten, G Ardine de Wit, Henk F van Stel, Michael F Gerhards, Marinus J Wiezer, Huib A Cense, Hein BAC Stockmann, Jeroen WA Leijtens, David DE Zimmerman, Eric Belgers, Bart A van Wagensveld, Eric DJA Sonneveld, Hubert A Prins, Peter PLO Coene, Tom M Karsten, Joost M Klaase, Markwin G Statius Muller, Rogier MPH Crolla, Ivo AMJ Broeders

**Affiliations:** 1 Department of Surgery, Meander Medical Center Amersfoort, Utrechtseweg 160, 3818 ES Amersfoort, The Netherlands; 2 Julius Center for Health Sciences and Primary Care, University Medical Center Utrecht, Heidelberglaan 100, 3584 CX Utrecht, the Netherlands; 3 Department of Gastroenterology, Meander Medical Center Amersfoort, Utrechtseweg 160, 3818 ES Amersfoort, the Netherlands; 4 Department of Surgery, Onze Lieve Vrouwe Gasthuis Amsterdam, Oosterpark 9, 1091 AC Amsterdam, the Netherlands; 5 Department of Surgery, Sint Antonius Hospital Nieuwegein, Koekoekslaan 1, 3435 CM Nieuwegein, the Netherlands; 6 Department of Surgery, Rode Kruis Hospital Beverwijk, Vondellaan 13, 1942 LE Beverwijk, the Netherlands; 7 Department of Surgery, Kennemer Gasthuis Haarlem, Boerhaavelaan 22, 2035 RC Haarlem, the Netherlands; 8 Department of Surgery, Laurentius Hospital Roermond, Mgr. Driessenstreet 6, 6043 CV Roermond, the Netherlands; 9 Department of Surgery, Diakonessenhuis Utrecht, Bosboomstraat 1, 3582 KE Utrecht, the Netherlands; 10 Department of Surgery, Atrium Medical Center Heerlen, Henri Dunantstraat 5, 6419 PC Heerlen, the Netherlands; 11 Department of Surgery, Lucas Andreas Hospital Amsterdam, Jan Tooropstraat 164, 1061 AE Amsterdam, the Netherlands; 12 Department of Surgery, Westfriesgasthuis Hoorn, Maelsonstraat 3,1624 NP Hoorn, the Netherlands; 13 Department of Surgery, Jeroen Bosch Hospital 's-Hertogenbosch, Tolbrugstraat 11, 5211 RW 's-Hertogenbosch, the Netherlands; 14 Department of Surgery, Maasstad Hospital Rotterdam, Olympiaweg 350,3078 HT Rotterdam, the Netherlands; 15 Department of Surgery, Reinier de Graaf Hospital Voorburg, Fonteynenburghlaan 5A, 2275 Voorburg, the Netherlands; 16 Department of Surgery, Medisch Spectrum Twente, Haaksbergerstraat 55, 7513 ER Enschede, the Netherlands; 17 Department of Surgery, MC Zuiderzee Hospital Lelystad, Ziekenhuisweg 100, 8233 AA Lelystad, the Netherlands; 18 Department of Surgery, Amphia Hospital Oosterhout, Pasteurlaan 9, 4900 AB Oosterhout, the Netherlands

## Abstract

**Background:**

Persisting abdominal complaints are common after an episode of diverticulitis treated conservatively. Furthermore, some patients develop frequent recurrences. These two groups of patients suffer greatly from their disease, as shown by impaired health related quality of life and increased costs due to multiple specialist consultations, pain medication and productivity losses.

Both conservative and operative management of patients with persisting abdominal complaints after an episode of diverticulitis and/or frequently recurring diverticulitis are applied. However, direct comparison by a randomised controlled trial is necessary to determine which is superior in relieving symptoms, optimising health related quality of life, minimising costs and preventing diverticulitis recurrences against acceptable morbidity and mortality associated with surgery or the occurrence of a complicated recurrence after conservative management.

We, therefore, constructed a randomised clinical trial comparing these two treatment strategies.

**Methods/design:**

The DIRECT trial is a multicenter randomised clinical trial. Patients (18-75 years) presenting themselves with persisting abdominal complaints after an episode of diverticulitis and/or three or more recurrences within 2 years will be included and randomised. Patients randomised for conservative treatment are treated according to the current daily practice (antibiotics, analgetics and/or expectant management). Patients randomised for elective resection will undergo an elective resection of the affected colon segment. Preferably, a laparoscopic approach is used.

The primary outcome is health related quality of life measured by the Gastro-intestinal Quality of Life Index, Short-Form 36, EQ-5D and a visual analogue scale for pain quantification. Secondary endpoints are morbidity, mortality and total costs. The total follow-up will be three years.

**Discussion:**

Considering the high incidence and the multicenter design of this study, it may be assumed that the number of patients needed for this study (n = 214), may be gathered within one and a half year.

Depending on the expertise and available equipment, we prefer to perform a laparoscopic resection on patients randomised for elective surgery. Should this be impossible, an open technique may be used as this also reflects the current situation.

**Trial Registration:**

(Trial register number: NTR1478)

## Background

The recurrence rate of patients treated conservatively for an episode of diverticulitis is approximately 25% [1]. Elective resection has traditionally been advised after a second episode of diverticulitis. It has been thought that patients with a diverticulitis recurrence are at greater risk of developing complications, have higher mortality rates and are less likely to respond to medical treatment [2].

However, recent studies have demonstrated that the number of attacks of diverticulitis is not necessarily a prevailing factor in defining the suitability of surgery. Most patients who present with complicated diverticulitis do so at the time of their first attack. Furthermore, only a fraction (5-7%) develops complicated diverticulitis during subsequent attacks [3,4]. This and the fact that operation itself carries significant morbidity and mortality, has lead to reluctance in gastroenterologists and surgeons towards elective resection after a recurrence of the disease.

However, elective resection may be an appropriate solution for a more selective group of patients who suffer greatly from their disease. Many studies have consistently shown that 40-80% remain symptomatic after conservative treatment, leading to impaired health-related quality of life (HRQoL) and increased costs due to multiple specialist consultations, pain medication and productivity losses [1]. Logically, this is also the case for patients who continue developing diverticulitis recurrences on a frequent basis. Also, these patients often remain symptomatic in between the recurrences.

In addition of possibly preventing further recurrences and complications of diverticulitis, elective resection has frequently been demonstrated to relieve persisting symptoms after an episode of diverticulitis [5,6]. Therefore, many physicians and patients seem to abandon expectant/conservative management and subsequently choose elective resection.

Both conservative and operative management of patients with persisting abdominal complaints after an episode of diverticulitis and/or frequently recurring diverticulitis are applied. However, direct comparison by a randomised controlled trial is necessary to determine which is superior in relieving symptoms, optimising HRQoL, minimising costs and preventing diverticulitis recurrences against acceptable morbidity and mortality associated with surgery or the occurrence of a complicated recurrence after conservative management.

## Methods

### Study objective

The DIRECT trial is a multicenter randomised clinical trial. The objective is to compare conservative management to elective resection of the diseased colon segment in patients with persisting abdominal complaints after an episode of diverticulitis and/or frequently recurring diverticulitis. We hypothesize that elective resection is superior in relieving abdominal complaints, preventing further hospitalisation and specialist consultation and minimising direct and indirect hospital costs against acceptable morbidity and mortality compared to conservative management.

### Study population

#### Inclusion criteria

- Age 18-75 years.

- Patients presenting with either persisting abdominal complaints and/or frequently recurring diverticulitis after a well documented (CT-scan or ultrasonography) episode of diverticulitis.

Persisting abdominal complaints may include patients with:

- continuing lower left abdominal pain AND/OR persistent change in bowel habits AND/OR persistent blood loss.

- Symptoms must exist longer than 3 months after a previous episode of diverticulitis

Frequently recurring diverticulitis is defined as:

- Three or more diverticulitis recurrences within 2 years.

- A minimal interval of 3 months between the recurrences is mandatory.

- Persisting abdominal complaints and/or frequently recurring diverticulitis must be accompanied by inflammatory changes (CT-scan or ultrasonography) in the bowel wall: Bowel-wall thickening with or without abscess.

- ASA I-III.

#### Exclusion criteria

- Patients with elective or emergency surgery for acute diverticulitis in the past.

- Patients with an absolute operation indication (perforation with purulent/fecal peritonitis, symptomatic bowel stenosis or fistula).

- Patients with colorectal malignancies.

- Patients in ASA class III who are at high risk for per- and postoperative complications due to severe co-morbidity as regarded by the surgeon and/or the patients specialists

- Patients with a psychiatric disease or other conditions making them incapable of filling out the questionnaires or completing the objective follow up tests.

### Study endpoints

#### Primary endpoint

Health-related quality of life (HRQoL) objectified primarily by the Gastro-intestinal Quality of life Index (GIQLI) and secondarily by EuroQol-5D (EQ-5D), Short-form 36 (SF-36) and Visual Analogue Score (VAS) for pain. Patients are also asked to point out on a 7 point Likert scale whether their health and complaints have improved or deteriorated in comparison to the previous assessment.

### Secondary endpoints

1. Mortality defined as:

○ *Elective surgery*: 30-days mortality.

○ *Both groups*: Mortality associated with the development of complications related to diverticulitis during follow-up.

2. Morbidity defined as:

○ Diverticulitis recurrence

○ Perforation (with purulent/fecal peritonitis)

○ Fistula

○ Symptomatic stenosis

○ Abscess

○ Stoma formation

○ Emergency surgery or re-operation

○ Peri- and postoperative complications

3. Direct health care costs. In-hospital resource use will be recorded. During follow-up medication use, general practitioner and specialist visits will be measured at baseline and regular intervals with customized questionnaires.

4. Indirect non-health care costs, using a standardised ShortForm-health and labour questionnaire (SF-H&L) at baseline and regular intervals during follow-up.

All questionnaires are asked to be filled in at baseline and 3, 6, 9, 12, 24 and 36 months after treatment

### Sample size

The sample size calculation is based on the minimum important difference* (MID) of the GIQLI score. The MID can be estimated by taking half a standard deviation of a quality of life instrument [7,8].

Based on the studies of Forgione et al and Zdichavsk et al the MID of the GIQLI score is estimated at 10 points [5,6]. They also demonstrated that patients improve with 10 points on the GIQLI score one month to one year after elective resection (111 ± 20.4 and 105.8 ± 15.5) for diverticulitis compared to preoperatively (100 ± 22.1 and 95.3 ± 21.4). In conclusion, a difference of 10 points corresponds with the MID and the expected improvement after elective resection.

To demonstrate this difference using an independent t-test (alpha = 0.05, delta = 10, sigma = 21, power = 0.9) approximately N = 97 patients per group are needed for this study. Therefore a total study population of 194 patients is required to attain statistical significance.

To compensate for a potential loss to follow-up of 10%, 214 patients will be included.

* Minimum important difference (MID): The smallest difference in score in the domain of interest which patients perceive as beneficial and which would mandate, in the absence of troublesome side effects and excessive cost, a change in the patient's (health care) management.

### Treatment of Subjects

#### Conservative treatment

Patients randomised for conservative treatment are treated according to the current daily practice. In other words, conservative treatment is determined by the preferences of the treating physician. Conservative treatment may consist of expectant management, antibiotics and/or analgetics. Should there be radiologic evidence for the presence of pericolic abscesses, percutaneous drainage may be performed depending on the size and opinion of the local radiologist regarding accessibility.

#### Elective surgery

Patients randomised for elective surgery will undergo an elective colonic resection within approximately 6 weeks. In the interval between randomisation and elective surgery, patients are treated conservatively (see above). Intentionally, a laparoscopic approach is used. The extent to which the colon is resected in the proximal direction should cover the entire macroscopically involved colon. In other words, the proximal resection line should be where no diverticula exist or at the level where a considerable decline in number of diverticula is noted. Distally, the margin of resection should be where the taenia coli splay out onto the upper rectum. After resection a primary anastomosis will be performed between the distal colon and rectum.

#### Randomisation (figure 1)

**Figure 1:**
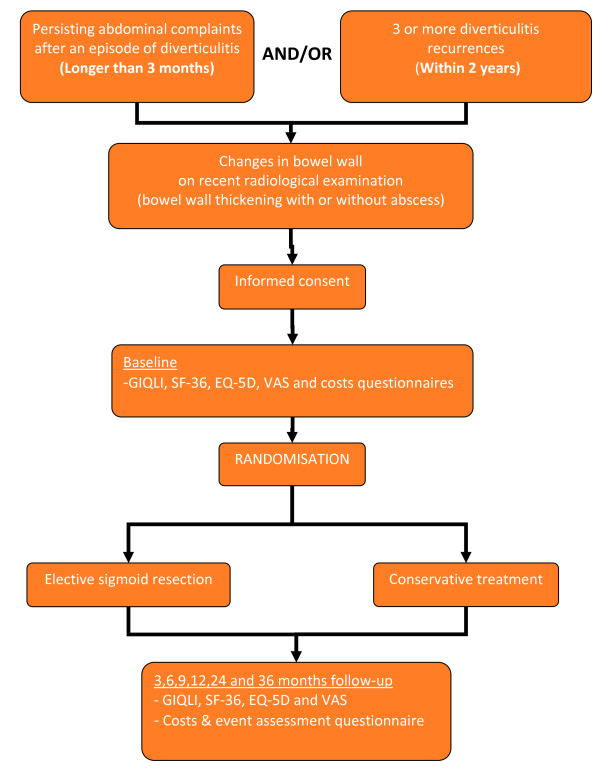
Flowchart DIRECT trial

All patients presenting themselves with persisting abdominal complaints after an episode of diverticulitis and/or a third (or more) diverticulitis recurrence, require to have had a recent radiological examination of the abdomen. Preferably a CT-scan is used. However, ultrasonography may also be used on the condition that bowel wall thickening and abscess size can be assessed accurately. Colonoscopy may be performed on indication to exclude malignancy.

If all inclusion criteria are met, patients are informed about the study protocol by their treating physician. They are given a 3 day reflective period, together with the information package. After the reflective period, the patient is contacted and asked for participation. If the patient decides to take part in the trial, he/she is invited to the local hospital to sign the informed consent. After receiving this consent form, randomisation will be performed centrally by the study coordinator using block randomisation (block size 6) stratified for center and inclusion criteria (persisting abdominal symptoms or frequent recurrences).

Both patients in the conservative and elective resection group will be treated conservatively in case of events during follow-up unless there is an absolute indication for surgery according to the treating physician (e.g. fistula, symptomatic stenosis, perforation with purulent/fecal peritonitis). In addition, in case of persisting abdominal symptoms during follow-up (in the conservative group) which are regarded as unbearable by both patient and treating physician, the treating physician may consult an independent event adjudication committee. The independent committee will advise the treating physician whether or not to abandon conservative management and proceed to elective resection. The final decision is made by the treating physician.

### Data collection

Data are collected by a local research fellow and/or treating physician at baseline, postoperatively (if randomised for elective resection), during outpatient visits and in case of adverse events leading to hospitalisation during follow-up. Case record forms on paper are used and faxed to the data manager.

Patients are asked to fill out HRQoL questionnaires at baseline. These questionnaires, as well as resource use and productivity losses questionnaires, are also sent to the patients at 3,6,9,12,24 and 36 months follow-up.

Data integrity will be checked when receiving the questionnaire. Any missing items will be collected by contacting the patient by telephone. Reminders (including a new copy of the questionnaire) will be send after two weeks.

### Statistical analysis

The statistical package SPSS will be used for analysis. All analyses will be performed according the intention to treat principle.

Baseline characteristics will be described as means and standard deviations. Large differences between treatment groups will be analysed with an independent Student's T-test to verify significance (p-value < 0.05). Significant differences will be adjusted for in the final analysis.

The primary outcome will be analysed using mixed linear models with random effects. The covariates of the random part of the model will be determined using restricted maximum likelihood estimation (REML) and selected on the basis of Akaike Information Criterium (AIC). For the fixed part, models will be constructed containing either the treatment effect adjusted for time with or without an interaction term of these components. The models will be compared using AIC. Missing data will be imputed using multiple imputation.

The estimates of the final model will be used to test whether or not there is a clinical difference between the treatment groups. As described before, the MID is 10 points on the GIQLI scale. Therefore, the MID will be subtracted from the estimated difference between treatment groups and tested with Wald's test against a p-value of 0.10.

Additionally, the 7 point scale reflecting self-reported improvement of complaints over time, will be used to confirm whether the assumption of MID being the equivalent of half a standard deviation, holds.

Categorical outcome measures will tested using the chi-square test (p-value < 0.05) and described as percentages and counts.

### Economic evaluation

The cost analysis will be performed form a societal perspective including total direct health care costs and indirect non-health care costs (productivity losses).

Direct health care costs include costs related to hospitalization, imaging, blood tests, colonoscopy, medication, interventions, operations, consultations, complications and primary health care contacts. On an individual patient basis, resource use will be recorded. Subsequently, by multiplying resource use with unit price, actual costs per patient will be calculated. Unit costs will be derived from the Dutch costing manual or determined in co-operation with hospital administration.

Health care consumption including general practitioner or specialist visits and medication use will be assessed using customised questionnaires and case report forms.

Indirect non-health care costs include sick leave from paid work, own expenses of patients and time and travel costs. Sick leave from paid work will be assessed using the ShortForm-Health and Labour questionnaire. The remaining indirect non-health costs will be assessed using customised questionnaires to be completed by participants.

The cost-effectiveness will be expressed as incremental costs per quality-adjusted life year (QALY) gained. QALY gains over time will be assessed using the EQ-5D classification system as completed by patients, in combination with pre-defined value sets for all possible health states. The time perspective of the analysis will be 3 years.

### Patient safety

After inclusion and completion of half a year follow-up of 25% of patients and after one year follow-up of 50% of patients in both groups, interim analysis will take place. A safety-monitoring committee consisting of independent physicians will review the results and advice the steering committee of the trial. The steering committee will decide on the continuation of the trial.

In addition all severe adverse events will be reported to the central Medical Ethics Committee and the independent safety-committee. The safety committee will discuss the events and will advice the trial steering-committee on the safety of the trial.

### Ethics

The study is conducted in accordance with the principles of the Declaration of Helsinki and "good clinical practice" guidelines. The protocol has been approved by the the medical ethical committee "Verenigde Commissies Mensgebonden Onderzoek", located at the St. Antonius Hospital, Nieuwegein, the Netherlands. Prior to randomisation, informed consent will be obtained form all patients.

## Discussion

Acute diverticulitis is diagnosed about 300 times alone at the department of surgery at the Meander Medical Center Amersfoort per year. A significant part consists of patients with persisting abdominal symptoms and frequent recurrences. Considering the high incidence and the multicenter design of this study, it may be assumed that the number of patients needed for this study (n = 214), may be gathered within one and a half year.

Depending on the expertise and available equipment, a laparoscopic approach is preferred for patients randomised for elective sigmoidresection. Preliminary results of the SIGMA trial have shown that elective laparoscopic sigmoid resection for diverticulitis leads to a better HRQoL compared to conventional resection. However, as this study aims to reflect the current situation (in which both conventional and laparoscopic approaches are used), conventional sigmoid resection may be used as an alternative.

## Abbreviations

HRQoL: Health-related Quality of Life; ASA: American Society of Anesthesiologists classification of preoperative risk; VAS: Visual Analogue Score; SF-36: Short Form-36; GIQLI: Gastrointestinal Quality of Life Index; SF-H&L: Short Form Health and Labour; MID: Minimum Important Difference; REML: Restricted maximum likelihood estimation; AIC: Akaike Information Criterium

## Competing interests

The authors declare that they have no competing interests.

## Authors' contributions

BJM and WA drafted the manuscript. ECJ and IAMJ co-authored the writing of the manuscript. All other authors and study groups participated in the design of the study during several meetings and/or are local investigators at the participating centers. All authors edited the manuscript and read and approved the final manuscript.

## Pre-publication history

The pre-publication history for this paper can be accessed here:

http://www.biomedcentral.com/1471-2482/10/25/prepub
